# Clinical manifestations and management of fatty acid oxidation disorders

**DOI:** 10.1007/s11154-020-09568-3

**Published:** 2020-07-11

**Authors:** J. Lawrence Merritt, Erin MacLeod, Agnieszka Jurecka, Bryan Hainline

**Affiliations:** 1 Pediatrics, University of Washington, Seattle, WA USA; 2 Children’s National Hospital, Washington, DC USA; 3 Ultragenyx Pharmaceutical Inc., Novato, CA USA; 4 Indiana University School of Medicine, Indianapolis, IN USA

**Keywords:** Fatty acid oxidation disorder, FAOD, Metabolism, Hypoglycemia, Cardiomyopathy, Rhabdomyolysis

## Abstract

Fatty acid oxidation disorders (FAOD) are a group of rare, autosomal recessive, metabolic disorders caused by variants of the genes for the enzymes and proteins involved in the transport and metabolism of fatty acids in the mitochondria. Those affected by FAOD are unable to convert fatty acids into tricarboxylic acid cycle intermediates such as acetyl-coenzyme A, resulting in decreased adenosine triphosphate and glucose for use as energy in a variety of high-energy–requiring organ systems. Signs and symptoms may manifest in infants but often also appear in adolescents or adults during times of increased metabolic demand, such as fasting, physiologic stress, and prolonged exercise. Patients with FAOD present with a highly heterogeneous clinical spectrum. The most common clinical presentations include hypoketotic hypoglycemia, liver dysfunction, cardiomyopathy, rhabdomyolysis, and skeletal myopathy, as well as peripheral neuropathy and retinopathy in some subtypes. Despite efforts to detect FAOD through newborn screening and manage patients early, symptom onset can be sudden and serious, even resulting in death. Therefore, it is critical to identify quickly and accurately the key signs and symptoms of patients with FAOD to manage metabolic decompensations and prevent serious comorbidities.

## Introduction

### Mitochondrial fatty acid oxidation and energy homeostasis

Energy needs vary markedly based on nutritional intake and exertion. Therefore, the ability to utilize different energy sources and store excess energy for later use is critical for maintaining proper homeostasis. Glucose is the primary fuel for the brain, whereas ketone bodies meet a large portion of energy needs of the skeletal muscle and heart [1, 2]. Energy is stored as glycogen in both the liver and skeletal muscle tissue, while proteins are stored in the muscle, and triglycerides are stored in adipose tissue [3].

**Fig. 1:**
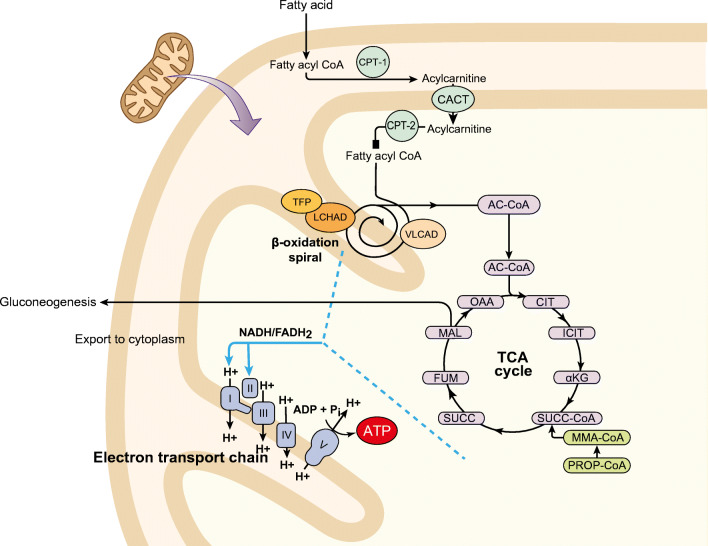
Role of key enzymes in the oxidation of fatty acids in the mitochondria. Adapted with permission from Vockley J, et al. Mol Genet Metab. 2015;116:53–60. αKG, α-ketoglutarate; AC-CoA, acyl-coenzyme A; ADP, adenosine diphosphate; ATP, adenosine triphosphate; CACT, carnitine acylcarnitine translocase; CIT, citrate synthase; CPT, carnitine palmitoyl transferase; FADH, flavin adenine dinucleotide; FUM, fumarase; ICIT, isocitrate dehydrogenase; LCHAD, long-chain L-3 hydroxyacyl-CoA dehydrogenase; MAL, malate dehydrogenase; MMA-CoA, methylmalonyl-CoA mutase; NADH, nicotinamide adenine dinucleotide; OAA, oxaloacetic acid; PROP-CoA, propionyl-CoA carboxylase; SUCC, succinate dehydrogenase; SUCC-CoA, succinyl-CoA synthetase; TCA, tricarboxylic acid; TFP, trifunctional protein; VLCAD, very-long-chain acyl-CoA dehydrogenase

During periods of decreased carbohydrate intake, prolonged fasting, or increased energy demands, the body’s glycogen stores are lessened or depleted (normal stores are estimated to be ~18 to 24 h of fasting for adults, less for small children, or about 100 min of exercise) [3, 4]. Under such circumstances, up to 80% of the energy needs of the heart, skeletal muscle, and liver are derived from the oxidation of fatty acids in healthy individuals, whereas this transformation is compromised in those with fatty acid oxidation disorders (FAOD) [5, 6]. The fatty acid oxidation process involves the release of fatty acids from adipose tissue (Fig. 1). Long-chain fatty acids and medium-chain fatty acids greater than 8 carbon atoms are transported into the mitochondria with the aid of L-carnitine, while short-chain fatty acids and medium-chain fatty acids up to 8 carbon atoms freely permeate the mitochondrial membrane via a carnitine-independent process [7]. Once inside the mitochondria, the breakdown of long-chain fatty acids, which are the main focus of this review, is characterized by three discrete stages. Stage 1 (β-oxidation) involves oxidation of long-chain fatty acids to yield acetyl residues in the form of acetyl-coenzyme A (CoA). In Stage 2, acetyl residues are oxidized to carbon dioxide via the tricarboxylic acid cycle. Finally, in Stage 3, electrons derived from the oxidation of Stages 1 and 2 are passed to oxygen via the mitochondrial respiratory chain for oxidative phosphorylation and adenosine triphosphate generation [8–10].

### Introduction to FAOD

FAOD are a group of rare, autosomal recessive, metabolic disorders stemming from variants in genes encoding any of ~20 enzymes and transport proteins utilized in fatty acid metabolism and transport via the carnitine shuttle for subsequent energy production by β-oxidation within the mitochondria [9, 11, 12], often with serious and potentially life-threatening consequences [13–15].

In patients with FAOD, reduced or loss of function of one of the mitochondrial proteins involved in fatty acid transport or metabolism can lead to tissue accumulation of fatty acids and/or their intermediate metabolites, as well as metabolic decompensation due primarily to the resulting depletion of tricarboxylic acid cycle intermediates such as acetyl-CoA [12].

There are several FAOD subtypes, which are classified primarily by the length of the fatty acid whose metabolism is disrupted or by the deficiency of the specific fatty acid transport protein. FAOD are diagnosed by analysis of plasma acylcarnitines or total and free carnitine levels (Table 1) and may be confirmed by gene sequencing. Estimates of FAOD incidence, as best predicted by identification from newborn screening (NBS), vary considerably based on subtype and geographic location (Table 1) [16].

**Table 1:** Approximate prevalence and acylcarnitine elevations of FAOD subtypes

	Gene	Prevalence [16]	Acylcarnitine elevations [17]
CPT-IAD	*CPT1A*	1:750,000 to 1:2,000,000	C0, C0/(C16 + C18) ratio
CACTD	*SLC25A20*		C16, C16:1, C18, C18:1
CPT-IID	*CPT2*		C16, C16:1, C18, C18:1
CTD	*SLC22A5*		C0
VLCADD	*ACADVL*	1:85,000	C12:1, C14:2, C14:1, C14, C16:1, C16
LCHADD	*HADHA*	1:250,000 to 1:750,000	C16:1-OH, C16-OH, C18:1-OH, C18-OH
TFPD	*HADHA, HADHB*		C16:1-OH, C16-OH, C18:1-OH, C18-OH
MCADD	*ACADM*	1:4000 to 1:15,000 to 1:200,000	C8, C10, C10:1
SCADD	*ACADS*	1:35,000 to 1:50,000	C4
MADD	*ETFA, ETFB, ETFDH*	rare	C4, C5, C6, C8, C10:1, C12, C14, C14:1, C16, C16:1, C18, C18:1, C16-OH, C16:1-OH, C18-OH, C18:1-OH

*C0* free carnitine, *CACTD* carnitine-acylcarnitine translocase deficiency, *CPT-IAD/CPT-IID* carnitine palmitoyltransferase I/II deficiency, *FAOD* fatty acid oxidation disorder, *LC-FAOD* long-chain fatty acid oxidation disorders, *LCHAD*D long-chain L-3 hydroxyacyl-CoA dehydrogenase deficiency, *MCADD* medium-chain acyl-CoA dehydrogenase deficiency, *TFPD* tri-functional protein deficiency, *VLCADD* very-long-chain acyl-CoA dehydrogenase deficiency

Carnitine transport system disorders, including carnitine-acylcarnitine translocase deficiency (CACTD), carnitine palmitoyltransferase I/II deficiency (CPT-IAD/CPT-IID), and carnitine uptake defect, are the least common FAOD subtypes, with a reported incidence of ~1:750,000 to 1:2,000,000. Among long-chain FAOD (LC-FAOD), including very-long-chain acyl-CoA dehydrogenase deficiency (VLCADD), long-chain L-3 hydroxyacyl-CoA dehydrogenase deficiency (LCHADD), and tri-functional protein deficiency (TFPD), the incidence varies markedly depending on the specific subtype, with VLCADD being the most common LC-FAOD (VLCADD, ~1:85,000; LCHADD/TFPD, ~1:250,000 to 1:750,000). The global incidence of medium-chain acyl-CoA dehydrogenase deficiency (MCADD) is ~1:4000 to 1:200,000; however, most countries report incidences of 1:4000 to 1:15,000 (Table 1) [16]. For example, the reported incidence of MCADD in Qatar is ~1:4000, whereas a study of patients in Australia, Germany, and the US reported an incidence of ~1:15,000 [16]. Taiwan reported the incidence of MCADD as 1:199,922 [18]. Finally, the incidence of short-chain acyl-CoA dehydrogenase deficiency has been reported at ~1:35,000 to 1:50,000 [19, 20]. Reports of the incidence of multiple acyl-CoA dehydrogenase deficiency (or glutaric aciduria II) also vary markedly (~1:15,000 to 1:2,000,000) [16].

**Fig. 2:**
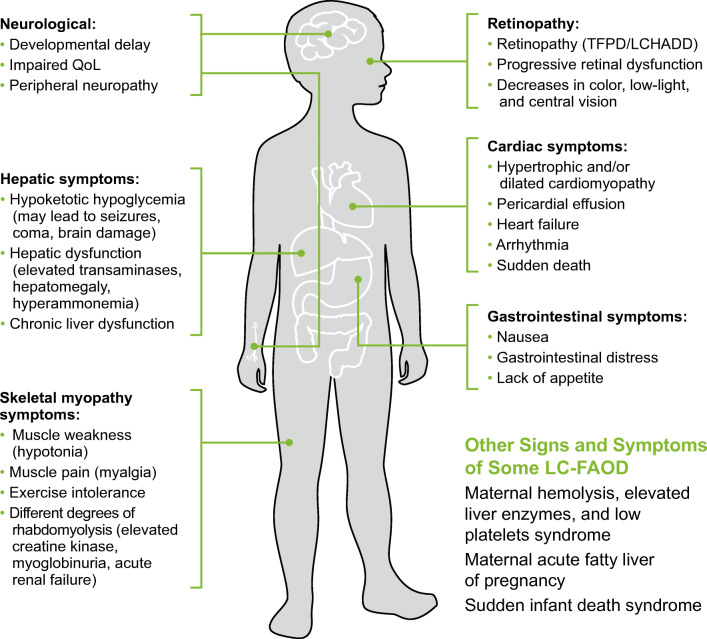
FAOD: clinical manifestations of disease can be serious, unpredictable, and precipitous in nature. Genotype-specific manifestations are denoted in parentheses, with others broadly applicable. FAOD, fatty acid oxidation disorder; LC-FAOD, long-chain fatty acid oxidation disorders; LCHADD, long-chain L-3 hydroxyacyl-CoA dehydrogenase deficiency; TFPD, tri-functional protein deficiency; QoL, quality of life.

The current review focuses primarily on the clinical manifestations associated with LC-FAOD and (to a lesser extent) MCADD, which affects a narrower group of organ systems compared with LC-FAOD. As a consequence of different enzymes contributing to energy production at different stages and through interaction with different substrates, the manifestations of disease, along with their timing, nature, and severity, can vary greatly (Fig. 2) [11]. LC-FAOD may present as acute metabolic crises during times of increased energy demand, such as common infections or moderate exercise; these metabolic crises can result in hypoglycemia, rhabdomyolysis, or cardiomyopathy [9, 12]. Onset can be unpredictable and sudden, and events may be life-threatening, requiring emergency medical intervention [9, 12]. Between crises, patients may have impaired exercise tolerance and their quality of life (QoL) may be poorer, for example, because of restrictions on physical activity and exercise due to fears of further metabolic crises [9, 21].

### Onset of FAOD

Initial onset of FAOD may occur early or late in life and is characterized by a broad spectrum of clinical disease presentations, affecting a variety of high-energy–requiring organ systems, including the heart, liver, and skeletal muscle and nervous systems. Symptom onset can occur at any time, from early infancy onwards, placing patients at serious risk of life-threatening episodes of spontaneous acute decompensation [11, 22]. In a series of 107 cases of inherited defects in fatty acid oxidation, symptoms manifested in 84% of patients before they were two years old, with approximately one-third first presenting as neonates [23]. A large proportion (46%) in this series presented with hypoketotic hypoglycemia; other symptoms reported in infants were coma triggered by fasting or catabolism, Reye Syndrome–like episodes, cardiomyopathy, and symptoms of acute myolysis [23]. Lack of symptoms in infancy or early childhood, however, does not mean that patients will be symptom free later. In one reported case, for example, despite being previously healthy, a 16-year-old girl developed recurrent exercise-induced rhabdomyolysis that resulted in eight hospitalizations [24]. In the case of a young man with suspected myocarditis, FAOD did not become clinically apparent until age 20 years [25]. Following intense physical activity, this patient experienced dyspnea, fatigue, myalgia, fever, and myoglobinuria. Worsening symptoms prompted an emergency department visit, at which an enlarged left ventricle was detected. The patient’s father died due to heart failure, and a sister died after prolonged recurrent illness. The patient was subsequently diagnosed with CPT-IID after genetic testing [25].

Overall, clinical evidence has demonstrated that the unpredictable nature of symptomology can make the diagnosis and management of FAOD challenging. Currently, management of FAOD typically involves dietary and lifestyle adjustment because no current FDA-approved treatment options are available. Additionally, treatment of the potentially life-threatening comorbidities (e.g., infections, fever, hypoglycemia, rhabdomyolysis) is essential to patient survival [26]. Such management can dramatically change the lives of patients with FAOD, as well as those of their parents and caregivers [27]. Careful consideration surrounds all aspects of patients’ lives because any change in nutrition, feeding schedule, or physical exertion could trigger a metabolic decompensation requiring hospitalization. Parents have referred to the management of their child’s disease as “the new normal” because the changes to daily life are so drastic [27].

### Detection of FAOD

Detecting and diagnosing FAOD can be difficult given that the timing of disease presentation can vary greatly from person-to-person, and may only be suspected following the onset of potentially deadly symptoms. One strategy to address this challenge is NBS, which has become increasingly available worldwide since being introduced in the late 1990s [16]. Most FAOD are identified by their specific acylcarnitine profiles using rapid acylcarnitine profile analysis using flow injection electrospray ionization tandem mass spectrometry (Table 1) [16, 28]. When combined with effective diagnostic algorithms designed to reduce this risk of false-positive signals, [29] NBS could enable efficient detection of LC-FAOD in individuals in whom symptoms alone might not have led to diagnosis.

Once the disease is identified, appropriate lifestyle precautions can result in improved clinical outcomes. However, in patients who do not undergo NBS or in those whose disease is not detected, unexpected serious outcomes can occur (e.g., death in up to 50% of patients diagnosed symptomatically) [16, 30]. In specific FAOD, identification by NBS and early disease management can have variable effectiveness in preventing symptom onset. Patients with VLCADD identified by NBS with milder reductions in enzymatic activity have shown decreases in frequency of hypoglycemic events as compared to historical reports from the pre-NBS era. In patients with dramatic reductions in enzymatic activity, hypoglycemia and cardiac symptoms could not be prevented despite early initiation of treatment following detection by NBS [31]. Of concern are findings from recent studies showing that despite NBS and prompt incorporation of nutritional management, early and serious manifestations, including death, can still occur [14]. Constant vigilance and subsequent recognition of key clinical manifestations are pivotal if rapid detection of any crises and implementation of appropriate management are to be achieved.

## General management of FAOD

Management of FAOD generally includes dietary restrictions to minimize reliance on long-chain fatty acid catabolism and, ultimately, prevent crises, although nutritional guidelines for FAOD vary depending on subtype and symptomatic or asymptomatic status.

Treatment recommendations for patients with more challenging subtypes, such as LCHADD/TFPD, suggest dietary protein and carbohydrate intake meet age-appropriate guidelines, whereas 20% of daily caloric intake should be derived from medium-chain triglyceride (MCT) [32, 33]. Dietary long-chain fats are significantly reduced while ensuring that levels of essential fatty acids, such as alpha-linoleic acid and linoleic fatty acids, are sufficient [32, 33]. Carnitine supplementation is also recommended to replace carnitine deficiency, although supplementation should be based on circulating plasma levels and excess carnitine should be avoided. When studied in the short-term, a higher-protein diet increased energy expenditure and decreased energy intake in patients with LCHADD/TFPD, suggesting that higher-protein diets may have benefit over higher-carbohydrate diets [34]. Nutrition recommendations for those with MCADD are to consume a generally heart-healthy diet. Dietary fat restriction is not indicated in patients with MCADD or LC-FAOD deficiencies with no or few symptoms; however, in highly symptomatic cases of LC-FAOD, long-chain fatty acids should be substituted by MCTs [35]. No specific dietary guidelines are currently published for CPT-IID, although treatment has followed similar recommendations as other LC-FAOD. Dietary management is typically based on an overall clinical assessment based on factors, including age of onset of symptoms, diagnosis, and presenting and ongoing symptoms. For example, patients with LC-FAOD manifesting symptoms in infancy may require highly restrictive dietary interventions, whereas those manifesting symptoms later in life may only require MCT supplementation with exercise.

Medium-chain triglyceride supplementation acts by bypassing any LC-FAOD defects, thereby allowing the body to process fatty acids normally. However, this can lead to an imbalance in the enzymes associated with the tricarboxylic acid cycle, warranting further supplementation. Notably, MCT is contraindicated in patients with MCADD. Timing of MCT supplementation throughout the day is essential for appropriate energy utilization. For example, specifically timing MCT supplementation prior to periods of exercise may improve energy availability and performance [36]. However, one alternative treatment under investigation is triheptanoin, an odd-carbon MCT comprising three 7-carbon fatty acids on a glycerol backbone. Unlike MCT, as well as acetyl-CoA, when metabolized, triheptanoin provides an additional energy source in the form of the 3-carbon propionyl-CoA, as well as 4- and 5-carbon ketone bodies [22]. Through an anaplerotic effect, this additional supplementation prevents depletion of key substrates, restoring energy production for gluconeogenesis [37]. Triheptanoin thus preserves tricarboxylic acid cycle function while bypassing the deficient enzymes.

Patients managing VLCADD with dietary long-chain fatty acid restrictions are at risk of deficiencies in both essential fatty acids and fat-soluble micronutrients [38]. These patients may require supplementation with docosahexaenoic acid or oils high in essential fatty acids, including linoleic acid and α-linoleic acid, to meet necessary nutritional needs [38]. Fasting depletes glycogen stores and forces the body to use fatty acids for energy; therefore, avoidance of fasting is key in the prevention of metabolic crises associated with FAOD. In neonates, additional precautions must be taken when weaning infants from breastfeeding or from an overnight feeding schedule [38].

Furthermore, Genetic Metabolic Dieticians International has recommended guidelines for younger patients with VLCADD. These guidelines note in asymptomatic patients, fasting should be limited to no more than 4 h in patients aged <4 months (with approximately one additional hour for each additional month of life), whereas symptomatic patients should have their maximum fasting period reduced by ~2 h from that recommended for asymptomatic patients. Similarly, according to Genetic Metabolic Dieticians International, healthy infants with MCADD should be fed at the same interval as those without MCADD (age < 4 months, maximum fasting of 4 h; age 5–12 months, maximum fasting of 4 h + 1 h per additional month of age) [39]. Moreover, treatment guidelines recommend a bedtime snack high in complex carbohydrates to prevent a metabolic decompensation overnight [38]. It is important patients prevent over-nutrition and excess weight gain because weight loss attempts can result in metabolic crisis.

Finally, patients often use personal lifestyle strategies, such as avoidance of physical activities, to minimize glucose depletion and prevent crises. Beyond the standard preventative management recommended for patients with FAOD, most treatments address specific symptom manifestations that arise during periods of decompensation. Parents of children with FAOD have reported that one of the most difficult aspects of being a caregiver is minimizing fasting by feeding their child every 3 h, as well as frequent trips to the grocery store to accommodate these altered meal plans [27]. This management strategy is challenging for both patients and their caregivers because strict dietary changes require constant vigilance and foresight. Some parents of children with FAOD have had to leave their jobs to adapt their lifestyles to better accommodate the complex treatment and management of FAOD. Particular challenges arise during holidays, vacations, or special events, when caregivers and patients must adapt their care routine to a new schedule or environment or when they have limited access to physicians or dietitians.

## Clinical manifestations of FAOD

FAOD give rise to a variety of clinical features, especially in organs that rely on energy production by fatty acid oxidation, such as the heart, liver, and skeletal muscle [9]. Historically, this led to FAOD research focusing predominantly on organ systems associated with the most serious outcomes including death, such as the heart and liver. Further research investigated other tissues such as skeletal muscle, which also can be profoundly affected by potentially life-threatening complications, such as rhabdomyolysis. Additional organ systems whose dysfunction generally is not life threatening are included in Table 2 [41–52]

**Table 2:** FAOD manifestations, age of onset, and presentation/symptoms

Manifestations	Contributing FAOD	Age of onset	Presentation/symptoms
Cardiac	• LC-FAOD [9]	• Neonatal or early childhood [11]	• Left ventricular wall hypertrophy may be observed initially and can progress to dilated cardiomyopathy with or without cardiac arrhythmia [40]
	- VLCADD	• May present later in life after periods of crisis [9]	• Sometimes accompanied by pericardial effusion
	- CPT-IID		• Sudden death
	- LCHADD		
	- TFPD		
Hepatic	• MCADD [9]	• Neonatal or early childhood [41]	• Reye-like symptoms:
	• LC-FAOD	• Typically within the first 2 years of life	• Hepatic encephalopathy and microvesicular steatosis of the liver and other tissues [12, 41]
	- CPT-IAD	• May present later in life after periods of crisis [12]	• Hypoketotic hypoglycemia
	- CPT-IID		• Hepatic dysfunction, characterized by jaundice, pale stools, enlarged liver, cholestasis (high bilirubin and c-GT, slight elevation of transaminases, normal platelet function), and axial hypotonia [23, 42]
	- LCHADD		• Signs of adrenergic symptoms and/or impairment of the nervous system, including lethargy, seizures, apnea, or coma [11, 43, 44]
	- CACTD		
Muscular	• All FAOD [9]	• Early childhood [45]	• Myalgia (muscle pain) [15, 26]
		• May present later in life provoked by endurance type activity, fasting, physiologic stress	• Hypotonia (muscle weakness)
			• Exercise intolerance
			• Myoglobinuria
			• Different degrees of rhabdomyolysis (ranging from subclinical rise of creatine kinase through myoglobinuria to acute renal failure)
Neurologic	• LC-FAOD [46]	• Neuropathy presents subtly and is not usually detectable until later in life (teens into adulthood) as the disease progresses [23, 47, 48]	• Slow, progressive sensorimotor polyneuropathy, along with limb-girdle myopathy with recurrent episodes of myoglobinuria
	- Generalized TFPD	• Neuropsychological manifestations are detectable earlier, as children miss key developmental milestones [49]	• Can present with autism spectrum disorders or intellectual disabilities [49, 50]
	- Isolated LCHADD		
	• All forms of FAOD have demonstrated links to intellectual disabilities		
Retinopathy	• LC-FAOD [46]	• Retinopathy is not usually evident until later in life [51]	• Patients experience progressive, irreversible vision loss, including decreased color vision, low-light vision, and vision in the center of the field of view [48]
	- Generalized TFPD	• Changes in the retina can be detected at around year 2	
	- Isolated LCHADD		
Other affected organ systems [52]:	• Lung: TFPD/CPT-IID	• Case reports have suggested respiratory distress in neonates	• Lung disease and respiratory distress have been reported anecdotally, and animal models with LC-FAOD have presented with altered breathing mechanics
	• Kidney: VLCADD/CPT-IID	• Reports have shown the potential for chronic kidney disease throughout life	• Renal cysts and fibrosis, typically seen in chronic end-stage kidney disease, have been reported in some patients
	• Immune: VLCADD	• Laboratory studies have suggested a potential link between FAOD and immune response	• Murine studies have indicated that FAOD may cause chronic, low-grade inflammation or an exaggerated immune response to pathogens

*c-GT* gamma-glutamyl transpeptidase, *CACTD* carnitine-acylcarnitine translocase deficiency, *CPT-IAD/CPT-IID* carnitine palmitoyltransferase I/II deficiency, *FAOD* fatty acid oxidation disorder, *LC-FAOD* long-chain fatty acid oxidation disorders, *LCHADD* long-chain L-3 hydroxyacyl-CoA dehydrogenase deficiency, *MCADD* medium-chain acyl-CoA dehydrogenase deficiency, *TFPD* tri-functional protein deficiency, *VLCADD* very-long-chain acyl-CoA dehydrogenase deficiency

### Cardiac manifestations

Fatty acids account for ~70% of the heart’s energy needs and, therefore, FAOD commonly manifest as cardiac dysfunction (Table 2) [1, 3, 10]. FAOD presenting with cardiac manifestations are most common in LCHADD, TFPD, neonatal CPT-IID, and VLCADD [9]; with rare reports in MCADD [53, 54].

#### Onset

The two primary categories for cardiac presentations stemming from FAOD are cardiomyopathies and cardiac arrhythmias. In patients with FAOD, cardiac conditions often appear during the neonatal period or in early childhood. When neonates experience acute periods of fasting (during weaning or from overnight feeds), their glycogen stores become depleted and they subsequently rely on fatty acid oxidation for energy, which triggers symptoms [35]. Some patients do manifest cardiac conditions later in life, although these events also typically occur under catabolic conditions, such as fasting, intense exercise, fever, or illness [9].

#### Pathophysiology

Patients with FAOD may develop dilated cardiomyopathy because they lack the enzymes required to break down fatty acids, which accumulate in the cytoplasm and lysosomes of cardiac tissue [40]. Subsequent misalignment of the cardiac myofibrils results in inefficient contraction of the heart, whereas toxic fatty acid accumulation in the cytoplasm and lysosomes of cardiac cells increases tissue size and inflammation. Hypertrophic cardiomyopathy occurs primarily due to insufficient energy availability in the heart tissue and subsequent inefficient contraction, thereby stimulating cardiac muscle hypertrophy [40, 55].

Cardiac arrhythmias in patients with FAOD stem from the inherent lack of energy production during those periods in which the body relies more heavily on fatty acid catabolism, such as the immediate postnatal period, or following intense exercise, fasting, or illness [6, 56]. The causes of these arrhythmias are often multifactorial, but they appear to primarily affect patients with LC-FAOD. Ventricular tachycardia can be caused by reduced single-channel conductance of inward-rectifier potassium channels, leading to automatic action potential discharges from resting and plateau potentials [57]. Conduction velocities are slowed by a decrease in the presence of excitatory sodium current, leading to re-entry arrhythmia [57]. Fatty acids that have not been esterified are able to directly activate the voltage-dependent calcium currents in cardiac myocytes, leading to cytotoxic calcium overload and subsequent arrhythmias due to oscillatory and nonoscillatory potentials [57, 58]. Finally, amphipathic metabolites impair gap-junction channels via disturbance of the cell membrane lipid-protein interface [57].

#### Signs and symptoms

Cardiac involvement is common in patients with FAOD, and these defects are a suspected cause of sudden death in early childhood [59]. Cardiomyopathies, alongside cardiac arrhythmias, commonly present in the immediate postnatal period [22]. The most common form of cardiomyopathy associated with FAOD is dilated cardiomyopathy, which can be asymptomatic or present as congestive heart failure, depending on the extent of ventricular dysfunction. In contrast, hypertrophic cardiomyopathies are typically more serious, with a higher risk of death [22].

Cardiac arrhythmias commonly present in the immediate postnatal period, when the demand for fatty acid oxidation is highest and frequently results in sudden infant death syndrome [57]. Many neonates with undetected/undiagnosed FAOD succumb to acute cardiac arrhythmia before any intervention is possible. Therefore, acute arrhythmia stemming from FAOD should always be considered in cases of sudden infant death syndrome [57]. However, it is important to acknowledge that cardiac arrhythmias can present at any age [22].

#### Management

For patients with cardiac symptoms stemming from FAOD, there are no approved targeted therapeutic options. Instead, current management options typically treat the presenting cardiac conditions (arrhythmia/cardiomyopathy). These currently available interventions include low-intensity aerobic exercise, β-blockers (secondary), calcium channel blockers (secondary), diuretics, vasoconstrictors, combination therapy (β-blockers or diuretics + anti-arrhythmic agents), and angiotensin-converting enzyme inhibitors [60]. In more highly symptomatic cases, or in those where damage from the disease has progressed, other interventions include inotropic therapy, respiratory ventilation, mechanical cardiac support, and heart transplant.

#### QoL impact

Poor cardiac function can lead to dizziness, diminished physical function, or anxiety over a potential cardiac event and may even require surgery or transplantation. Following the development of cardiomyopathy or arrhythmia, patients require additional lifestyle changes, including low-intensity aerobic exercise [60]. Patients with cardiomyopathy and arrhythmia, regardless of cause, have reported decreased physical and psychological well-being [61, 62].

### Hepatic manifestations

Another of the more common presentations for patients with FAOD is liver dysfunction, affecting the majority of patients who present with LC-FAOD and in those with MCADD during crises (Table 2) [11, 12, 57]. Hepatic manifestations of FAOD are most common in MCADD and LC-FAOD, including VLCADD, LCHADD, infantile CPT-IID and CPT-IAD, and CACTD [9].

#### Onset

Liver dysfunction secondary to FAOD typically occurs early in life and presents in varying degrees of severity, ranging from episodic hypoketotic hypoglycemia to mild liver dysfunction to severe liver disease or Reye-like syndrome [12, 41]. Presentation of hepatic manifestations also occurs during periods of crisis later in life [12].

#### Pathophysiology

One mechanism by which energy stores are replenished is the conversion of fatty acids to ketone bodies in the liver, which can then serve as an energy source for other tissues, including skeletal muscle, the heart, and the brain [5, 63]. When glucose stores are depleted, the body releases fatty acids from adipose tissue for energy. However, the inability of the liver to metabolize fatty acids results in steatosis, whereby fatty acids accumulate in the liver, and the subsequent decreased production of ketones [63].

From birth, overnight fasting promotes lipolysis and ketogenesis; however, in children with FAOD, impaired β-oxidation impacts ketone production and dependence on glycogen stores. The subsequent depletion of glycogen stores manifests as hypoketotic hypoglycemia in which children present with hypoglycemia without an accompanying increase in serum ketones, which is a key indicator in the evaluation of patients with hypoglycemia and suspected FAOD [64, 65].

#### Signs and symptoms

In children, initial symptoms of hepatic-presenting FAOD are commonly classified as Reye-like, which includes hypoketotic hypoglycemia, hepatic encephalopathy, hepatomegaly, hyperammonemia, microvesicular steatosis of the liver and other tissues, or unexplained hepatic failure, although true hepatic failure is less common [23].

The inability to break down fatty acids released from adipose tissue to replenish energy stores leads to a hypoglycemic state [3]. A key distinguishing characteristic of the form of hypoglycemia experienced by patients with FAOD is a lack of serum ketone bodies [43, 64]. In neonates, the presentation of hypoketotic hypoglycemia secondary to FAOD can vary; however, it typically exhibits some signs of adrenergic stimulation and/or impairment of the central nervous system in the form of lethargy, seizures, apnea, or coma [11, 43, 44].

Acute episodes of hypoglycemia during overnight infant fasting, when FAOD are unsuspected, may present as sudden death [11, 64]. FAOD should be considered as an underlying factor in any infants who die by sudden infant death syndrome [66]. Many FAOD manifesting as hypoglycemia stem from defects in the MCADD pathway; however, if accompanied by the cardiac defects (as detailed above), then LC-FAOD should also be considered [64]. Symptoms of liver dysfunction can include jaundice, pale stools, enlarged liver, cholestasis (increased bilirubin and glutamyl transferase, slight elevation of transaminases, normal platelet function), and axial hypotonia [23].

#### Management

Among patients who experience an acute hypoglycemic episode, adherence to a low blood glucose protocol may reverse the effects by restoring free glucose levels [11]. This may require intravenous glucose at 2 mL/kg in infants or up to 12–15 mg/kg/min in older children and adults. The main goal of long-term treatment of hypoketotic hypoglycemia secondary to FAOD is to stop the attempted catabolism of fatty acids.

Standard interventions for children with FAOD and hepatic crisis include avoidance of fatty acid oxidation inhibitors (e.g., valproic acid, nonsteroidal anti-inflammatory agents, and salicylates), non-use of intravenous lipid emulsions, and initiation of L-carnitine therapy (50 mg/kg/day via enteral or intravenous administration) [67]. Furthermore, symptoms may improve following the switch to a diet low in long-chain fatty acids, along with MCT supplementation [23]. Hepatic manifestations typically resolve within one month of returning to a strict nutritional plan, including minimal long-chain fats and supplementation with MCTs [23]. Surgical management should include the avoidance of agents such as propofol and lactate-containing fluids, which place stress on the mitochondria and encourage fatty acid utilization. These therapeutic approaches have led to some improvements in short-term outcomes, with some remaining risk of hypoglycemia and muscle symptoms may still occur; in the longer-term, risks still include non-hepatic consequences such as retinitis pigmentosa and neuropathy [23, 37].

Acute hypoglycemic episodes frequently result in hospitalization, and patients undergoing medical examinations or procedures that require extended fasting (>8 h) must be hospitalized before the procedure as a precautionary measure [35]. A further complication is the difficulty in avoiding hypoglycemia while traveling, particularly when crossing time zones, because meal times and food availability can be disrupted [68].

#### QoL impact

The impact of hypoketotic hypoglycemia on patient and caregiver QoL is similar for those with or without an FAOD as the primary cause. Acute episodes can result in patients experiencing mood swings (including irritability, stubbornness, and depression) with recurrent episodes causing feelings of powerlessness, anxiety, and depression in both patients and their families [68]. Children may also be admitted to the hospital (frequently for episodes without hypoglycemia, but with altered oral intake and behavior issues from illness). Such episodes place stress on the family units, especially if they occur during the first years of life.

Children and adolescents with hepatic steatosis have reported diminished QoL, including increased anxiety, depression, and low self-esteem [69]. Furthermore, hepatic encephalopathy has been associated with impairment in daily functioning, learning ability, sleep disturbances, and an increased risk of falls warranting hospitalization [70].

### Skeletal myopathy

Fatty acids are the principal source of energy for resting muscle, accounting for 85% of its energy needs [2]. During exercise, the primary energy source of skeletal muscle varies by exercise intensity, duration, and type, with maximal fatty acid oxidation occurring at moderate exercise intensity [5]. Glycogen stores are typically depleted following exercise, depending on the duration and intensity, thereby forcing the body to rely on fatty acid catabolism for energy [3, 4]. When the fatty acids cannot be catabolized effectively, chronic muscle damage may occur with increased risk of rhabdomyolysis in cases of acute energy deficiency. FAOD presenting with skeletal myopathy are most common in LCHADD, TFPD, VLCADD, and later-onset CPT-IID [9].

#### Onset

In contrast to the manifestations of FAOD that present in neonates, the skeletal myopathy symptoms of FAOD tend to usually appear in older individuals, from toddlers and older children through to adults, typically starting in puberty or adulthood. Toddlers may show early symptoms of muscle impairment during development of motor skills and walking [45]. Alternatively, symptoms can appear later in life following periods of endurance-type exercise, fasting, or physiologic stress (e.g., anesthesia, viral illness, emotional stress, cold exposure, sleep deprivation) [45].

#### Pathophysiology

Elevated plasma creatine kinase and myoglobin, for example following prolonged exercise, are indicative of rhabdomyolysis and suggestive of possible underlying FAOD [21]. Currently, the pathogenesis of rhabdomyolysis among individuals with FAOD is poorly understood [71]. However, it is known that rhabdomyolysis can be triggered by periods of fasting or exercise, resulting in deficient delivery of adenosine triphosphate to the muscle cells, which disrupts cellular integrity, ultimately leading to cellular disintegration [72]. In FAOD, depleted glycogen stores in conjunction with the inability to process fatty acids results in deficiency of adenosine triphosphate in muscle cells leading to rhabdomyolysis [45]. The consequent disintegration of striated muscle results in the release of sarcomere-specific constituents, such as myoglobin, into the extracellular fluid and circulation. Normally, myoglobin is loosely bound to plasma globulins and only small amounts reach the urine. However, when massive amounts of myoglobin are released, the binding capacity of the plasma protein is exceeded. Rhabdomyolysis may range from subclinical creatine kinase elevation through muscular weakness and myoglobinuria to medical emergency due to interstitial and muscle cell edema, contraction of intravascular volume, and pigment-induced acute renal failure [72]. Decreased muscle contraction leads to the muscle weakness and symptoms such as the disrupted gait commonly associated with FAOD [26]. There are no therapies to prevent the onset of rhabdomyolysis associated with FAOD, and patients with known FAOD are usually advised to avoid intense or prolonged exercise [71]. Patients without a diagnosis of FAOD who present with atypical rhabdomyolysis (i.e., muscular weakness and myoglobinuria are frequent, or cases where the preceding activities do not typically cause rhabdomyolysis in healthy individuals) should be tested for FAOD [72].

#### Signs and symptoms

In children or toddlers, the symptoms of early muscular disease that appear include hypotonia, disrupted gait, and muscle weakness. As the disease progresses, progressive or episodic muscle weakness, muscle pain (myalgia), chronic fatigue, exercise intolerance, and rhabdomyolysis may occur [15, 26]. Patients with FAOD who present with rhabdomyolysis have reported generalized muscle pain in conjunction with dark urine, most commonly following periods of intense exercise [21].

#### Management

The primary management of patients experiencing skeletal muscular manifestations of FAOD is avoidance of aggravating symptoms that trigger crises. This includes adherence to the usual nutritional guidelines and lifestyle changes, such as avoidance of excess activity [73]. The use of additional MCT oil supplementation, with appropriate dosage and timing in relation to activity, may allow patients to have an active, healthy lifestyle while encouraging the building of lean body mass [36]. Lean body mass may also be preserved by encouraging a diet higher in protein (25% total energy needs) than is typically recommended [74].

#### QoL impact

Muscular symptoms can significantly impact patients’ QoL and activities of daily living, with some patients reporting difficulty climbing stairs, walking, opening doors, or even supporting their own weight [26]. Such muscular symptoms can have a profound impact not only on children by hindering their ability to play or participate in sports, but also on adults who may find it difficult to engage in regular daily activities or lose weight. Because of muscle pain with exertion, many patients are sedentary, and exercise-induced rhabdomyolysis frequently leads to exercise avoidance. Evaluation of QoL in one clinical trial, using SF-36 v2 physical composite scores, highlighted the impact of limited physical activity because of myopathy [75]. Additionally, muscular symptoms in conjunction with the restricted diet and limited fasting can contribute to an increased risk of obesity [73].

### Neurological manifestations

Neurological symptoms (including both peripheral neuropathies and neuropsychological outcomes) have been reported in patients with FAOD but these are less common than cardiac, hypoglycemic, or muscular symptoms (Table 2). These neuropathies are most prevalent in specific forms of FAOD, including TFPD and LCHADD. Peripheral neuropathy occurs in ~80% of patients with TFPD and in 5–10% of patients with LCHADD [46]. It occurs later in life (teens or into adulthood), typically presenting as a slow, progressive sensorimotor polyneuropathy, along with limb-girdle myopathy with recurrent episodes of myoglobinuria [47].

#### Onset

Neuropsychological symptoms are typically more subtle than some of the more severe physical manifestations that are experienced by individuals with FAOD. Delays in neurological milestones emerge later in a child’s development (teens or into adulthood) [12, 49]. Various FAOD subtypes can often present similar symptoms, whereas others display a distinct genotype-phenotype correlation.

#### Pathophysiology

The underlying mechanisms associated with the development of neurological and neuropsychological disorders secondary to FAOD remain unclear. However, it is known that energy deficiencies, unmetabolized intermediates, and docosahexaenoic acid deficiencies can all hinder brain development and may lead to some or all of the cognitive damage associated with these observed outcomes [50]. Importantly, LC-FAOD can also be associated with low docosahexaenoic acid levels due to dietary over-restrictions. Avoidance of over-restriction and supplementation is advocated [48]. This is an area that has not been explored extensively.

#### Signs and symptoms

Several studies have assessed the neuropsychological outcomes of patients diagnosed with FAOD of varying subtypes. Patients with MCADD and LC-FAOD have presented with signs and symptoms that raise concerns over their neurological development, such as speech deficits; motor, language, or learning issues; or requirement for early intervention services [49]. Additional studies have also reported autism spectrum disorders stemming from specific LC-FAOD. Furthermore, some patients with specific LC-FAOD present with intellectual disability [50].

There have been additional reports of episodes of chronic, progressive peripheral neuropathy in patients, which occur in those individuals afflicted specifically with LCHADD or TFPD [47, 48]. Of concern, these neuropathies are irreversible despite current treatment efforts [46]. For example, peripheral neuropathies cannot be corrected using high-caloric intake, and progressive and acute neurologic symptoms are not always associated with the more easily detectable hypoglycemia [12].

#### Management

Neuropathy stemming from FAOD is irreversible and can be progressive; however, the pathophysiology of this manifestation is not well understood. Consequently, no specific management guidelines are available beyond lifestyle changes and the recommended long-chain triglyceride restrictions associated with other LC-FAOD [46]. While there are no specific guidelines for neuropathies stemming from FAOD, common treatments for peripheral neuropathy include physical and occupational therapies to manage pain and loss of function [76].

#### QoL impact

The onset of peripheral neuropathy can vary based on underlying FAOD; however, it persists as a progressive presentation of the disease. As the neuropathy progresses, it can require more frequent clinical monitoring, although its treatment can be difficult [17]. Moreover, since this manifestation is irreversible, it poses a lifelong impact on patient QoL [46].

### Retinopathy

Despite their primary reliance on glucose for energy, the cells of the retina have been shown to also express enzymes of the β-oxidation pathway. Defects of these enzymes in retinal cells lead to pigmentary retinopathy and ultimately vision loss (Table 2) [51]. Retinopathies stemming from FAOD are most common in LCHADD and TFPD [46]. Retinopathies affect >30–50% of patients with LCHADD and 5–13% of those with TFPD [46].

#### Onset

Some patients with FAOD develop vision problems, which typically present later than other manifestations. Of those patients with vision loss, ~50% have evidence of retinal changes by age 2 years [51]. Notably, once these changes to the retina have started, the damage is progressive and irreversible despite current treatment efforts [46].

#### Pathophysiology

The pathophysiology of retinopathy secondary to FAOD has yet to be elucidated; however, several theories have been proposed. Findings from studies have indicated that the number of metabolic decompensations is positively correlated with vision loss in patients with FAOD [51], suggesting that elevated levels of metabolic by-products during these crises may cause the observed retinopathy. A different theory has implicated a mutant α-subunit of the TFP as the driver of retinal cell death, leading to vision loss [51]. A further putative mechanism underlying the development of retinopathy involves the elevated plasma levels of hydroxyacylcarnitines and hydroxy fatty acids reported during metabolic crises that may have a role in the destruction of retinal cells [51].

#### Signs and symptoms

Chronic and progressing retinopathy stemming from FAOD has been reported to manifest in four stages [66]. Stage 1 is characterized by a normal retina with hypopigmented fundus, whereas Stage 2 features the appearance of pigment clumping in the fovea with progressive retinal dysfunction (age-appropriate vision is unaffected). In Stage 3, the central pigmentation disappears and chorioretinal atrophy leads to macular pallor (noticeable night and color vision loss occurs). Finally, Stage 4 involves the posterior pole of the eye being devoid of photoreceptors, and most central vision is lost. Overall, patients’ experience of retinopathy secondary to FAOD includes decreases in color vision, low-light vision, and vision in the center of the field of view [48].

#### Management

It is known that adherence to an FAOD-specific nutrition plan reduces the levels of hydroxyacylcarnitines and hydroxy fatty acids and slows the progression of vision loss. Therefore, a strict diet combined with MCT supplementation is recommended to slow the progression of retinopathy. Although docosahexaenoic acid supplementation has not been shown to prevent the progression of retinal damage, visual acuity improvements have been noted. Supplementation of L-carnitine has not demonstrated any benefits in patients with retinopathy secondary to FAOD [48].

#### QoL impact

The aforementioned patient experience of retinopathy in terms of decreased color vision, low-light vision, and vision in the center of the field of view inevitably makes tasks such as reading and driving more difficult, particularly in low-light conditions [48]. The diminished QoL associated with vision loss secondary to general retinopathy, not necessarily associated with FAOD, has been attributed to several factors, such as reduced income and economic security, reduced social interaction and support, and a diminished sense of control over one’s life, including a greater reliance on others [77].

## Family planning and pregnancy

Although the outcomes of FAOD can be serious, it is also important to consider the potential effects of these disorders on women who are pregnant. It has been noted that the human placenta expresses six of the enzymes of the β-oxidation pathway at levels similar to those of skeletal muscle. It is acknowledged that certain disorders affecting fatty acid oxidation can have adverse effects on women during pregnancy [78]. For example, some mothers of children born with LCHADD or TFPD develop acute fatty liver of pregnancy or maternal hemolysis, elevated liver enzymes, and low platelets syndrome [78, 79].

Mothers heterozygous for FAOD and pregnant with an affected fetus can develop preeclampsia, acute fatty liver of pregnancy, maternal hemolysis, elevated liver enzymes, and low platelets syndrome and can deliver a premature, intrauterine growth–restricted child. In pregnancies of fetuses with LCHADD or general TFPD, maternal liver disease occurs 20–70% of the time [79]. Furthermore, 62% of mothers developed acute fatty liver of pregnancy or maternal hemolysis, elevated liver enzymes, and low platelets syndrome in pregnancies of fetuses with LCHADD [79]. Among women pregnant with a fetus with a FAOD, neither the fetus nor the placenta are able to fully oxidize fatty acids, resulting in transfer of metabolic intermediates to the maternal circulation [79]. It is believed that these intermediates are what leads to preeclampsia, maternal hemolysis, elevated liver enzymes, low platelets syndrome, and acute fatty liver of pregnancy.

## Key conclusions

FAOD are a group of rare, potentially life-threatening autosomal-recessive disorders that affect a wide range of organ systems and can be unpredictable in the timing and severity of their onset. Moreover, identifying a genotype-phenotype link is not always clear, making it difficult to predict outcomes (Table 2) [22]. However, it does appear that certain genetic variants, namely LC-FAOD, are associated with a greater propensity for decompensation events in affected individuals [14].

Currently, there are only limited management options for this array of disorders aside from strict adherence to nutritional plans to minimize crises [11]. Without proper identification based on NBS or symptoms, patients cannot be instructed to follow the recommended nutritional plan, leading to dysfunction that can be chronic, progressive, and fatal. Importantly, although NBS may be effective in reducing certain events in patients with FAOD (e.g., hypoglycemic events in patients with residual enzyme activity), serious clinical symptoms (e.g., cardiac symptoms) are known to manifest rapidly and unpredictably, and death can occur despite appropriate management [14, 31]. Recognition of clinical manifestations is crucial in the timely identification and management of crises stemming from FAOD.

## Abbreviations

FAOD: fatty acid oxidation disorder
CoA: coenzyme A
NBS: newborn screening
CACTD: carnitine-acylcarnitine translocase deficiency
CPT-IAD: carnitine palmitoyltransferase I deficiency
CPT-IID: carnitine palmitoyltransferase II deficiency
LC-FAOD: long-chain fatty acid oxidation disorder
VLCADD: very-long-chain acyl-CoA dehydrogenase deficiency
LCHADD: long-chain L-3 hydroxyacyl-CoA dehydrogenase deficiency
TFPD: tri-functional protein deficiency
MCADD: medium-chain acyl-CoA dehydrogenase deficiency
QoL: quality of life
MCT: medium-chain triglyceride
